# Supplementary material for: Molecular mechanism of gallium nitrate in inhibiting bacterial biofilm formation through pykF modulation
Source: PLoS One. 2026 Mar 6;21(3):e0337557. doi: 10.1371/journal.pone.0337557 (PMC12965525; doi:10.1371/journal.pone.0337557)
Supplement: S1 Fig — Concentrations tested included 4096 μg/ml, 2048 μg/ml, 1024 μg/ml, 512 μg/ml, 256 μg/ml, 128 μg/ml, 64 μg/ml, 32 μg/ml, and 8 μg/ml. The bacterial suspension was diluted with gallium nitrate at 1:1000 to yield a final concentration of 1 × 10^5^ CFU/ml. After incubation at 37°C for 24 hours, cultures were photographed and examined. (DOCX) [file pone.0337557.s001.docx]

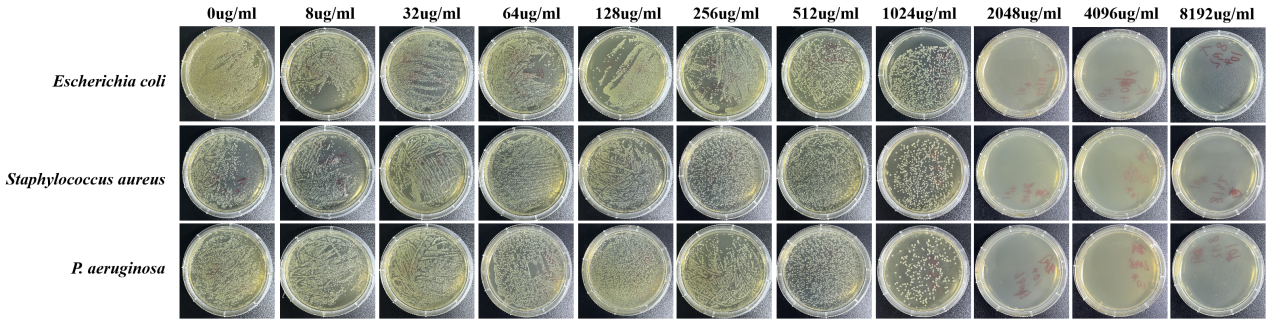


Supplementary Figure 1. Screening of the optimal GA intervention concentration. Concentrations tested included 4096 μg/ml, 2048 μg/ml, 1024 μg/ml, 512 μg/ml, 256 μg/ml, 128 μg/ml, 64 μg/ml, 32 μg/ml, and 8 μg/ml. The bacterial suspension was diluted with gallium nitrate at 1:1000 to yield a final concentration of 1×10^5^ CFU/ml. After incubation at 37°C for 24 hours, cultures were photographed and examined.
