# Supplementary material for: Molecular mechanism of gallium nitrate in inhibiting bacterial biofilm formation through pykF modulation
Source: PLoS One. 2026 Mar 6;21(3):e0337557. doi: 10.1371/journal.pone.0337557 (PMC12965525; doi:10.1371/journal.pone.0337557)
Supplement: S2 Fig — (B) Combined effects of gallium nitrate (GA) exposure and pykF deletion on pykF expression. (a) RT-PCR analysis of pykF mRNA levels in E. coli with rpoA as internal reference. The relative fold changes were as follows: KO-pykF vs. KO-NC, 0.552; KO-pykF + GA vs. KO-NC + GA, 0.279; KO-pykF + GA vs. KO-pykF, 1.094. (b) Western blot analysis of pykF protein expression with GAPDH as internal control. Relative fold changes were: KO-pykF vs. KO-NC, 0.250; KO-pykF + GA vs. KO-NC + GA, 0.474; KO-pykF + GA vs. KO-pykF, 2.570. Significance levels: *P < 0.05, **P < 0.01, ***P < 0.001, ****P < 0.0001. (DOCX) [file pone.0337557.s002.docx]

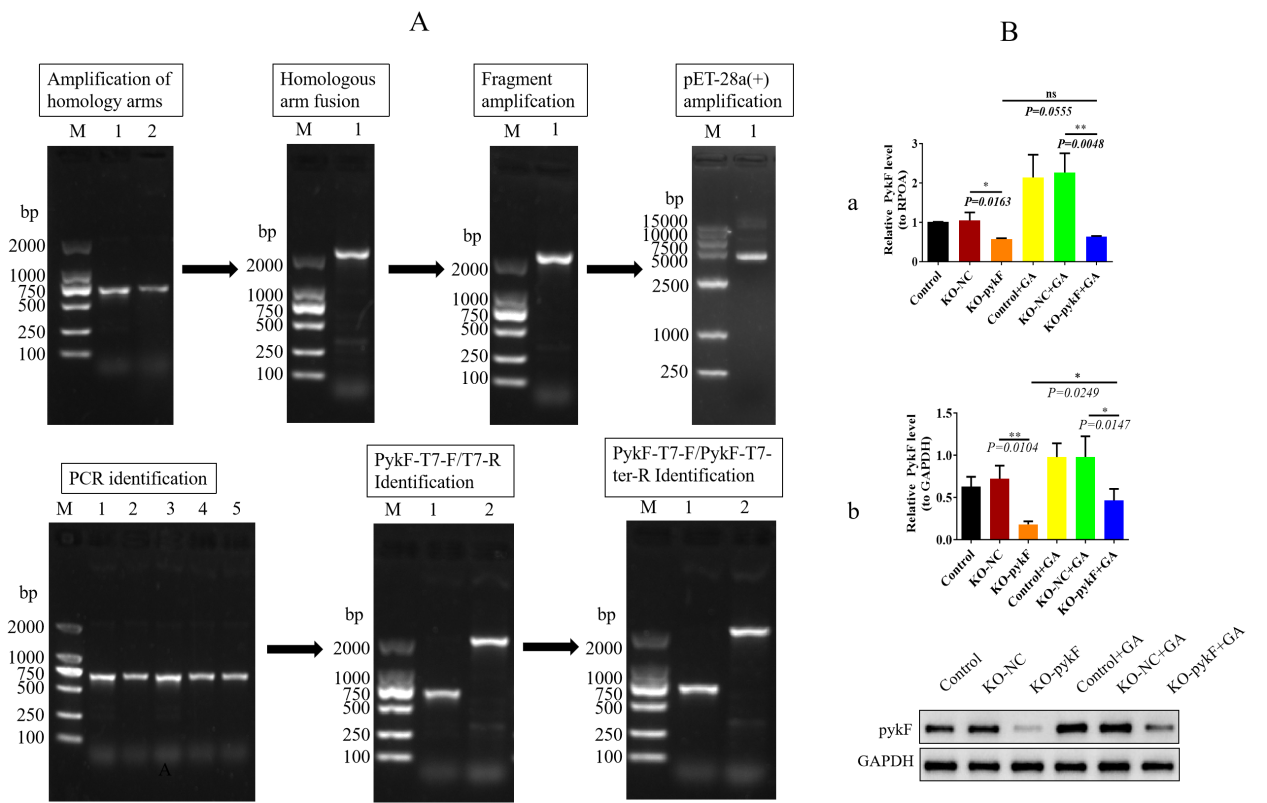


Supplementary Figure 2. (A) Strategy for generating the *pykF* knockout (KO) strain in *E. coli*. (B) Combined effects of gallium nitrate (GA) exposure and *pykF* deletion on *pykF* expression. (a) RT-PCR analysis of *pykF* mRNA levels in *E. coli* with rpoA as internal reference. The relative fold changes were as follows: KO-*pykF* vs. *KO-NC*, 0.552; *KO-pykF*+GA vs. *KO-NC*+GA, 0.279; *KO-pykF*+GA vs. *KO-pykF*, 1.094. (b) Western blot analysis of *pykF* protein expression with GAPDH as internal control. Relative fold changes were: *KO-pykF* vs. *KO-NC*, 0.250; *KO-pykF*+GA vs. *KO-NC*+GA, 0.474; *KO-pykF*+GA vs. *KO-pykF*, 2.570. Significance levels: *P < 0.05, **P < 0.01, ***P < 0.001, ****P < 0.0001.
