# Supplementary material for: Molecular mechanism of gallium nitrate in inhibiting bacterial biofilm formation through pykF modulation
Source: PLoS One. 2026 Mar 6;21(3):e0337557. doi: 10.1371/journal.pone.0337557 (PMC12965525; doi:10.1371/journal.pone.0337557)
Supplement: S3 Fig — (A) Experimental timeline: bacteria cultured at week 0; animal modeling at week 1; body weight and temperature monitored weekly through week 10; animals maintained until week 12, when euthanasia and retrieval of polydimethylsiloxane implants were performed. (B) Culture outcomes of WT-E. coli and KO-pykF-E. coli groups. (C-a) Tibial modeling procedure. (C-b) Subcutaneous dorsal modeling procedure. (D) Longitudinal changes in body weight (left) and temperature (right) of rats infected with WT-E. coli or KO-pykF-E. coli from weeks 0–10. (E) Polydimethylsiloxane implants collected from dorsal subcutaneous sites of rats in WT-E. coli and KO-pykF-E. coli groups. (DOCX) [file pone.0337557.s003.docx]

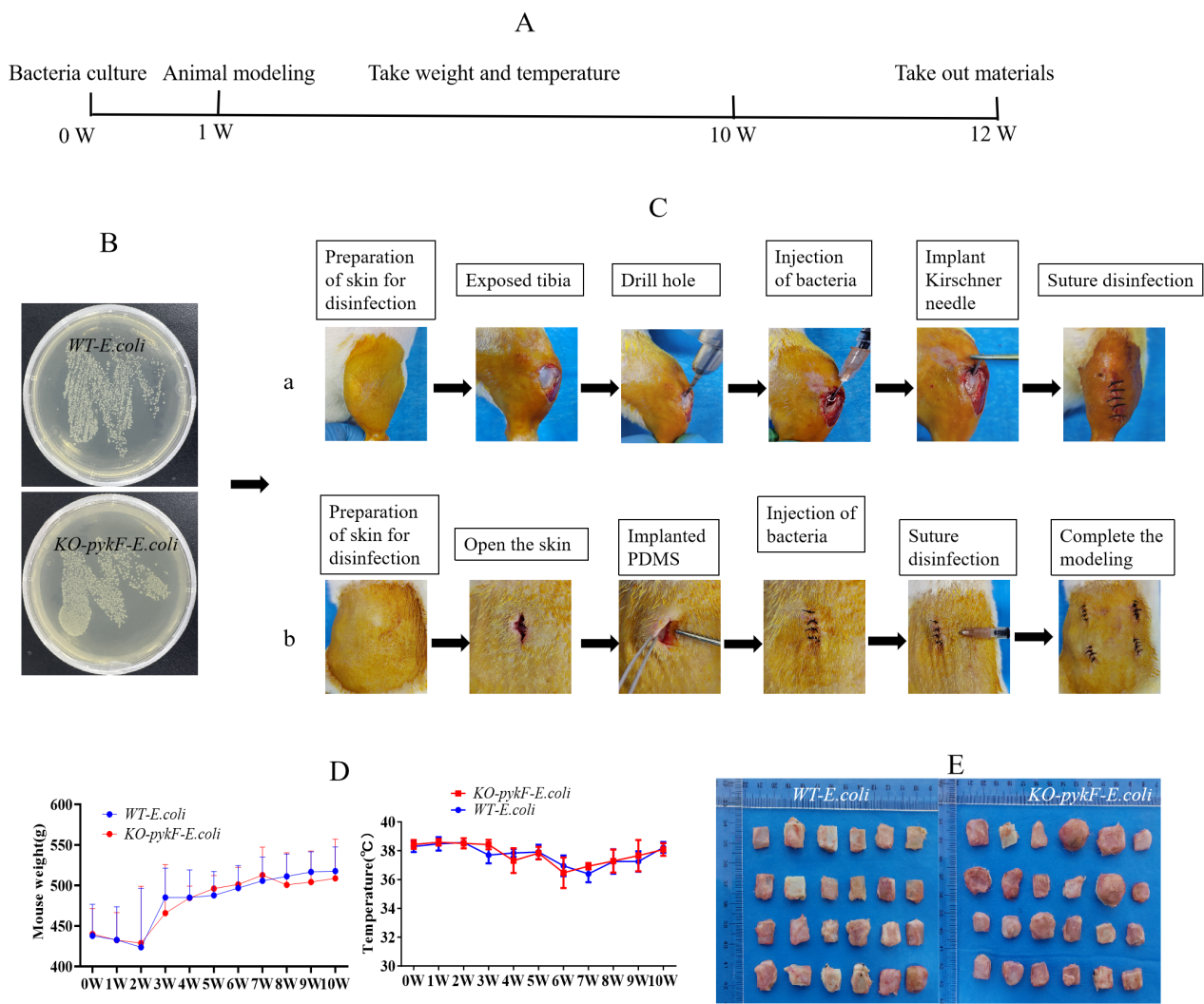


Supplementary Figure 3. *In vivo* evaluation of *pykF* in biofilm formation using a rat bone infection model. (A) Experimental timeline: bacteria cultured at week 0; animal modeling at week 1; body weight and temperature monitored weekly through week 10; animals maintained until week 12, when euthanasia and retrieval of polydimethylsiloxane implants were performed. (B) Culture outcomes of WT-*E. coli* and *KO*-*pykF*-*E. coli* groups. (C-a) Tibial modeling procedure. (C-b) Subcutaneous dorsal modeling procedure. (D) Longitudinal changes in body weight (left) and temperature (right) of rats infected with WT-*E. coli* or *KO*-*pykF*-*E. coli* from weeks 0–10. (E) Polydimethylsiloxane implants collected from dorsal subcutaneous sites of rats in WT-*E. coli* and *KO*-*pykF*-*E. coli* groups.
