# Supplementary material for: Molecular mechanism of gallium nitrate in inhibiting bacterial biofilm formation through pykF modulation
Source: PLoS One. 2026 Mar 6;21(3):e0337557. doi: 10.1371/journal.pone.0337557 (PMC12965525; doi:10.1371/journal.pone.0337557)
Supplement: Attachment — Submitted filename: Peer Review Report - PONE-D-25-24459.docx [file pone.0337557.s004.docx]

**Peer Review Report**

**Manuscript Title**: *Molecular mechanism of gallium nitrate in inhibiting bacterial biofilm formation through pykF modulation*
**Manuscript Number**: PONE-D-25-24459
**Recommendation**: *Minor Revision*

**General Assessment**

This manuscript presents a comprehensive investigation into the antimicrobial mechanisms of gallium nitrate, highlighting the *pykF* gene as a central molecular target in biofilm inhibition. The authors employ a well-integrated experimental design, including transcriptomics, in vitro and in vivo models, molecular docking, and functional validation. The study addresses a clinically relevant issue — biofilm-associated infections on implant surfaces — and proposes a novel and promising therapeutic avenue. Overall, the work is scientifically sound and well-aligned with the scope of PLOS ONE.

**Major Strengths**

1. **Scientific Rigor**:
   The methodology is robust, combining transcriptomic screening with functional assays (qPCR, Western blot, CLSM, SEM, animal infection models), providing strong evidence for the role of *pykF* in gallium nitrate's mode of action.
2. **Relevance and Originality**:
   The identification of *pykF* as a regulatory target of gallium nitrate in biofilm suppression is novel and of translational value, especially in the context of implant-related infections.
3. **Data Presentation**:
   The results are clearly presented and supported by appropriate statistical analyses and high-quality figures.

**Points for Minor Revision**

1. **Language and Grammar**:
   - The manuscript requires substantial English editing to correct awkward phrasing and enhance clarity.
   - Examples of unclear sentences:
     - "In regulation gallium nitrate, a nonredox iron III analog, inhibits bacterial biofilms..."
     - "Gallium nitrate treatment similarly inhibited biofilm formation in both wild-type and pykF-knockout strains." (ambiguous interpretation)
   - Suggest using a professional English editing service prior to acceptance.
2. **Discussion Improvements**:
   - The discussion would benefit from a deeper comparison with existing literature regarding other metal-based antibiofilm agents and alternative *pykF* regulatory mechanisms in various bacterial species.
3. **Limitations Section**:
   - The limitations are only briefly mentioned. The authors should expand this section by addressing the short observation period, the lack of human data, and the restricted range of bacterial species tested.
4. **Data Availability**:
   - While the authors state that data are fully available within the manuscript and supplementary files, depositing raw transcriptomic data in a public repository (e.g., GEO, FigShare) with a DOI would align better with PLOS ONE’s data policy.
5. **Statistical Analysis**:
   - Please clarify whether normality was tested and which post hoc tests were applied in the ANOVA analyses.

**Conclusion**

This is a well-conceived and timely study that makes a meaningful contribution to the field of antimicrobial research. With minor revisions to language, discussion depth, and data accessibility, the manuscript will be suitable for publication.
